# The evolution and maintenance of trioecy with cytoplasmic male sterility

**DOI:** 10.1038/s41437-024-00729-7

**Published:** 2024-10-14

**Authors:** M. T. Nguyen, J. R. Pannell

**Affiliations:** Department of Ecology and Evolution, University of Lausanne, 1015 Lausanne, Switzerland

**Keywords:** Population genetics, Plant reproduction, Cytogenetics

## Abstract

Trioecy, the co-existence of females, males and hermaphrodites, is a rare sexual system in plants that may be an intermediate state in transitions between hermaphroditism and dioecy. Previous models have identified pollen limitation as a necessary condition for the evolution of trioecy from hermaphroditism. In these models, the seed-production and pollen production of females and males relative to those of hermaphrodites, respectively, are compromised by self-fertilization by hermaphrodites under pollen- limitation. Here, we investigate the evolution of trioecy via the invasion of cytoplasmic male sterility (CMS) into androdioecious populations in which hermaphrodites co-occur with males and where the male determiner is linked to a (partial) fertility restorer. We show that the presence of males in a population renders invasion by CMS more difficult. However, the presence of males also facilitates the maintenance of trioecy even in the absence of pollen limitation by negative frequency-dependent selection, because males reduce the transmission of CMS by females by siring sons (which cannot transmit CMS). We discuss our results in light of empirical observations of trioecy in plants and its potential role in the evolution of dioecy.

## Introduction

Plants are remarkable for the diversity of polymorphic sexual systems (Barrett 2002; Pannell and Jordan 2022). These range from hermaphroditism (where all individuals transmit genes through both male and female functions) through androdioecy or gynodioecy (where hermaphrodites co-occur with males or females, respectively) to dioecy (where individuals are either male or female) (Pannell and Jordan 2022). In theory, we might also expect to find the maintenance in some populations of all three sex-allocation strategies—males, females and hermaphrodites. However, this sexual system, often labelled ‘trioecy’ (Sakai and Weller 1999; Ehlers and Bataillon 2007), is strikingly rare in plants (Yampolsky and Yampolsky 1922; Godin 2022), though it can be invoked as a possible intermediate state in transitions between hermaphroditism and dioecy (Charlesworth and Charlesworth 1978; Bawa 1980). In particular, theory for the evolution of dioecy from hermaphroditism (Charlesworth and Charlesworth 1978) supposes such transitions are likely to begin with the evolution of gynodioecy or androdioecy via the spread of a sterility mutation that transforms hermaphrodites into females or males, respectively, and that, in turn, a second sterility mutation may invade the population, converting the remaining hermaphrodites into the opposite sex. This scenario would thus involve a transition through subdioecy (Delph and Wolf 2005; Ehlers and Bataillon 2007), in which males and females coexist with ‘leaky’ plants that produce an arbitrary number of flowers of the other sex (Baker and Cox 1984; Humeau et al. 1999, Venkatasamy et al. 2007: Wang et al. 2014, Cossard and Pannell 2019), or trioecy, where males and females coexist with a third discrete class of hermaphrodites (Charlesworth and Charlesworth 1978; Charlesworth 1999).

There has been only limited analysis of the conditions allowing the evolution and maintenance of trioecy. In an evolutionarily stable strategy model of the evolution of sex-allocation polymorphisms, Charnov et al. (1976) showed that trioecy would not be stable in cases where the sexual phenotypes are due to nuclear genes and reproductive resources are shared between the sexes via a trade-off. Ross and Weir (1976) analysed a model with implicit biparental inheritance of sex-allocation phenotypes and also concluded that trioecy could not easily be maintained: they showed that hermaphrodites can only co-exist with males and females when the two sterility loci are unlinked, so that hermaphrodites could segregate as a result of recombination between them—but neuters would then also be present. Maurice and Fleming (1995) presented the first model that showed the stability of trioecy, specifically under conditions in which seed production is pollen-limited, compromising the fitness of unisexual individuals, and hermaphrodites can be maintained because of their ability to self-fertilize. On the basis of a different model, however, Wolf and Takebayashi (2004) concluded that pollen limitation would not result in stable trioecy, but the reasons for the difference between the two models’ predictions were not elaborated.

The evolution of a sex-allocation polymorphism such as trioecy ultimately requires the invasion of new sexual phenotypes into a population that have higher fitness when rare than the more common resident phenotype(s) (Fisher 1930; Ayala and Campbell 1974). Importantly, evaluation of the relative fitness of the respective phenotypes depends on whether the genetic variants responsible are transmitted maternally or biparentally. A male-sterility mutation at a nuclear locus (with biparental inheritance) can invade a fully outcrossing hermaphroditic population if the resulting females produce more than twice the number of surviving seed progeny of the hermaphrodites (i.e., *g* > 2, where *g* is the seed production of females compared with hermaphrodites) (Lewis 1941; Lloyd 1974a, 1974b, 1975; Charlesworth and Charlesworth 1978). In contrast, the same male-sterility phenotype caused by a mutation in the mitochondrial genome (and thus transmitted uniparentally, usually through ovules) can invade a hermaphroditic population if the resulting females produce just slightly more seeds than hermaphrodites (i.e., *g* > 1) (Lewis 1941; Lloyd 1974a, 1974b, 1975). In the former case, the invasion of females leads to a gynodioecious equilibrium, with the frequency of females < 0.5, whereas, in the latter, the frequency of females can reach high frequencies and could theoretically fix in the population, tantamount to driving it to extinction (Lewis 1941; Lloyd 1974a, 1974b, 1975; Charlesworth and Charlesworth 1978; Charlesworth and Ganders 1979; Charlesworth 1981). Clearly, in the latter scenario trioecy could only evolve via gynodioecy if males invade a population before it becomes extinct.

To date, all models investigating the evolution of trioecy have assumed nuclear sterility mutations, but the role of cytoplasmic male sterility in transitions from hermaphroditism to gynodioecy is well-established, both theoretically (Jacobs and Wade 2003) and empirically. CMS can arise spontaneously and has been useful as an aid to crop breeding in agriculture (Edwardson 1956; Chen and Liu 2014). CMS has also been studied in detail in several gynodioecious species, including *Silene vulgaris* (Olson and McCauley 2002), *Silene acaulis* (Städler and Delph 2002)*, Thymus vulgaris* (Belhassen et al. 1991), Planta*go lanceolata* (De Haan et al. 1997) and many others (Kaul 1988). These studies have contributed to testing theoretical predictions concerning the relative seed production of females and hermaphrodites, selfing rates of the hermaphrodites and inbreeding depression.

Theoretical work of the evolution of gynodioecy with CMS was first published by Lewis (1941), who drew attention to the lower female seed production required for the invasion of CMS into a hermaphroditic population than in the case of gynodioecy due to nuclear male sterility. Since then, the invasion of CMS into hermaphroditic populations has been extensively explored and refined. This theory includes the dynamics of gynodioecy when CMS is countered by a fertility restorer mutation at a nuclear locus (Charlesworth and Ganders 1979; Charlesworth 1981; Delannay et al. 1981; Dufaÿ et al. 2007), the interactions of multiple CMS and restorer loci (Ross and Gregorius 1985; Gouyon et al. 1991), and the complexities of spatial dynamics (Frank 1989; Couvet et al. 1998; Frank and Barr 2001; Dufay and Pannell 2010). Schulz (1994) considered the evolution of dioecy via the invasion of males into populations segregating for CMS, but he did not pay much attention to potential trioecious intermediates.

To our knowledge, no model has yet considered the question of how the presence of males in a population might modify CMS invasion dynamics. Because androdioecy is rare, we should not expect the invasion of CMS into populations with males and hermaphrodites to be able to occur very often. However, androdioecy does occur in several species, including at least one case, polyploid *Mercurialis annua*, in which cytoplasmic male sterility also appears to be common (Nguyen et al. 2024) and which thus demands an explanation. Trioecy in *M. annua* was first described by Perry et al. (2012) for a few populations in southeastern Spain and was recently documented by Nguyen et al. (2024) as being widespread across the Iberian Peninsula. Apart from mapping its distribution across Spain and Portugal, Nguyen et al. (2024) also estimated the relative fitness components for males, females and hermaphrodites and inferred a cytoplasmic basis for male sterility. At present, there are no models that would allow us to predict or account for the observed variation in sex morphs in trioecious populations *M. annua*, notably the consistently low frequency of females. The invasion of CMS into a population already segregating for males is also relevant to our general understanding of the evolution of combined versus separate sexes and the possible role of intermediates that involve cytoplasmic male sterility.

In this paper, we introduce three versions of a new model to explore the conditions that allow the invasion of CMS into hermaphroditic and androdioecious populations as well as the conditions for the maintenance of trioecy, androdioecy and gynodioecy. In all three models, we considered the invasion of males and females into a hermaphroditic population, allowing us to compare and verify our models with the current literature (Lloyd 1975, Charlesworth and Charlesworth 1978); the invasion of females into an androdioecious population, providing the insight into the role of males on CMS dynamics; and conditions for the potential maintenance of trioecy. The models are also relevant to the maintenance of leaky dioecy, where the males and females allocate only a limited amount of their resources to the female and male functions, respectively. Each model considers the evolution and maintenance of the respective sexual systems as a function of the relative male and female fecundities, self-fertilization by hermaphrodites and potential inbreeding depression suffered by selfed progeny, contrasting modes of pollen-limited seed production, and the genetic architecture of male and female sterility and male-fertility restoration. Model 1 considers the impact of pollen limitation on seed production by females, while Models 2 and 3 consider two different instances of pollen limitation. We also used simulations to investigate the invasion of males into a gynodioecious population, allowing us to draw comparisons with the previous models of Schultz (1994) and Maurice et al. (1994).

## Methods

### Sex-allocation phenotypes and genotypes

Parameters used in our models are summarised and defined in Table 1. We assume that self-fertile hermaphrodites produce *G*_h_ ovules and *P*_h_ pollen grains and males produce *P*_m_ = *αP*_h_ pollen grains, with *α* > 1 reflecting the reallocation of resources from seed production to increased pollen production via a sex-allocation tradeoff. Sex is determined at a locus with disomic inheritance, with males expressing a dominant female-sterility allele, i.e., we assume an XY sex-determination system in which males and hermaphrodites have genotypes XY and XX. XY sex determination has been described for several androdioecious plants e.g., *Datisca glomerata* (Wolf et al. 2001), *M. annua* (Pannell 1997a), and Sagittaria lancif*olia* (Muenchow 1998) (reviewed in Pannell 2002). Both hermaphrodites and males may carry a CMS mutation that reduces male fertility. Specifically, hermaphrodites with the CMS mutation (i.e., the female phenotype) produce no pollen and *gG*_h_ ovules, with *g* > 1 reflecting a sex-allocation tradeoff, whereas males produce (1 – *e*_m_)*P*_m_ = (1 – *e*_m_)*αP*_h_ pollen, i.e., the effect of CMS on males may be partially reversed by the Y allele, which thus acts as a partial male-fertility restorer. The parameter *e*_m_ here models the effect of the CMS mutation on males, with *e*_m_ = 0 reflecting the case where the male function of males carrying the CMS mutation is fully restored (so that they produce the normal amount of pollen) and *e*_m_ = 1 reflecting the case where the male-fertility restorer has epistatic expression, functioning in hermaphrodites but not males (so that males with the CMS mutation are fully sterile and produce no pollen).

**Table 1:** Parameters of the models and their biological meanings.

Parameter	Biological meaning
*g*	The ratio of a female’s seed production to that of a hermaphrodite
$\alpha$	The ratio of a male’s pollen production to that of a hermaphrodite
*s*	Prior selfing rate of a hermaphrodite
*d*	Proportion of selfed seeds aborted due to inbreeding depression
*e*_m_	Proportion of a male’s pollen aborted due to CMS
*G*_h_	A hermaphrodite’s seed production
*P*_h_	A hermaphrodite’s pollen production
*P*	Average pollen production of one individual in the population
*P*_X_	Proportion of pollen carrying the X chromosome

### Pollen limitation and the mating system

We modelled three different scenarios with respect to the interaction of pollen limitation and the selfing rate. In all three models, hermaphrodites self-fertilize a proportion *s* of their ovules prior to outcrossing, irrespective of the amount of outcross pollen in the population. Model 1 assumes no pollen limitation, so the mating system is fully described by the rate of prior selfing, *s*, whereas Models 2 and 3 both assume pollen-limited seed production.

In Models 2 and 3, ovules not destined for prior-selfing may fail to be fertilized if there are fewer than *P*_h_ pollen grains in the population per individual (averaged over all hermaphrodites, males and females). Specifically, we assume that the probability that non-prior-selfed ovules will be outcrossed is equal to min(1, *P*/*P*_h_), where *P* is the average pollen produced per individual, averaged over all individuals. This assumption means that seed production in fully hermaphroditic populations is not pollen-limited. The models further assume that the invasion of CMS into a hermaphroditic population will bring about pollen limitation in proportion to the frequency of females, so that, whenever there are no males in the population, a population with only females (i.e., hermaphrodites expressing the CMS mutation) would fail to produce any seeds (pollen limitation would be complete). Previous modelling has shown that such a situation can bring about population extinction in the absence of nuclear fertility restoration alleles (Lewis 1941; Lloyd 1974a, 1974b, 1975). Models 2 and 3 both assume that pollen limitation limits the number of fertilized outcrossed seeds produced by females and hermaphrodites, such that their outcrossed seed production equals min(1, *P*/*P*_h_)*gG*_h_ and min(1, *P*/*P*_h_)*G*_h_, respectively. Model 2 assumes that ovules that fail to be fertilized by outcrossing simply abort in females and hermaphrodites, whereas Model 3 assumes that hermaphrodites self-fertilize these ovules via ‘delayed’ selfing. All self-fertilized ovules (whether by prior or delayed selfing) suffer inbreeding depression, with a fraction *d* failing to reproduce.

### Recurrence equations

We assumed non-overlapping generations for all models. For each generation, the pollen haplotype frequencies were calculated by summing the contributions of each genotype, using its frequency, its pollen production and Mendelian segregation ratios. Ovule haplotypes were calculated similarly. Zygote frequencies for the next generation were calculated based on the ovule and pollen frequencies, accounting for the death of a fraction *d* of self-fertilized zygotes, as described above. Our recurrence equations follow the same logic as those in the model of Delannay et al. (1981). The recurrence equations for the three models are

$$\begin{array}{lll}{f}_{{\rm{XX}},{\rm{n}}}^{t+1} \, = \, \frac{{O}_{{\rm{XX}},{\rm{n}}}^{t}{P}_{{\rm{X}}}^{t}+(S{1}_{{\rm{XX}},{\rm{n}}}^{t}+S{2}_{{\rm{XX}},{\rm{n}}}^{t})(1-d)}{{N}_{S}^{t}}\\ {f}_{{\rm{XY}},{\rm{n}}}^{t+1} \, = \, \frac{{O}_{{\rm{XX}},{\rm{n}}}^{t}{P}_{{\rm{Y}}}^{t}}{{N}_{S}^{t}}\\ {f}_{{\rm{XX}},{\rm{c}}}^{t+1} \, = \, \frac{{O}_{{\rm{XX}},{\rm{c}}}^{t}{P}_{{\rm{X}}}^{t}}{{N}_{S}^{t}}\\ {f}_{{\rm{XY}},{\rm{c}}}^{t+1} \, = \, \frac{{O}_{{\rm{XX}},{\rm{c}}}^{t}{P}_{{\rm{Y}}}^{t}}{{N}_{S}^{t}}\end{array}$$

where *f*^*t*^_*i,j*_ is the frequency of the genotype with sex chromosome *i* and cytotype *j* at time *t*, with *j* = n for the non-CMS cytotype and c for the CMS cytotype. Similarly, *O, S*1 and *S*2 refer to the number fertilized of outcrossing ovules, prior selfed ovules and delayed selfed ovules, respectively, with superscript and subscript defined as for *f*. *P*^*t*^_X_ and *P*^*t*^_Y_ are the proportions of pollen with sex chromosomes X and Y, respectively, at time *t*. *N*^*t*^_S_ is the total number of viable zygotes produced at time *t*. These recursion equations are presented in detail for each model in the Supplementary Information A.1.2.2 and the Mathematica scripts.

### Stability analysis

In addition to our main calculations based on fitness comparisons (Supplementary Information, sections A.1, A.2), we also determined criteria for the invasion and fixation of CMS in a population on the basis of the eigenvalues of a Jacobian matrix of partial derivatives that relate allele-frequency change to small perturbations from an equilibrium; if all the eigenvalues of the matrix have negative Real parts, the equilibrium is locally stable (see Otto and Day 2007, p. 294-237). We analysed the Jacobian matrix for each of our three models in Mathematica 13.0, with each row of the matrix containing the four partial derivatives of the CMS frequency as a function of the frequency of each of the four genotypes in the system. CMS should be unable to invade when the leading eigenvalue < 1 when there are no females or males carrying CMS in the population, whereas it should remain fixed when the leading eigenvalue < 1 for a population without hermaphrodites. The results we obtained from these analyses are identical to those calculated by comparing cytotype fitnesses, presented below as Eqs. (1)–(6).

### Simulations

We simulated the model starting with a population of hermaphrodites fixed for the X chromosome and cytotype n. To establish an androdioecious population at equilibrium, males (i.e., genotype XY, n) were introduced at a low frequency (10^−6^) and allowed to evolve to equilibrium. Females (i.e., genotype XX, c) were then introduced at low frequency (10^−6^) and the population was allowed to evolve to equilibrium. In both phases, populations were deemed to have reached equilibrium when genotype frequencies changed less than 10^−10^ in 20 generations. For the scenarios in which males invaded a gynodioecious population, we simply reversed the order in which unisexual individuals were introduced. In cases where CMS was destined to fix, theoretically leading to population extinction (e.g., in models with no pollen limitation), we introduced the invasion of males prior to extinction when the CMS frequency reached 0.99.

## Results

### Overview of the results

**Fig. 1:**
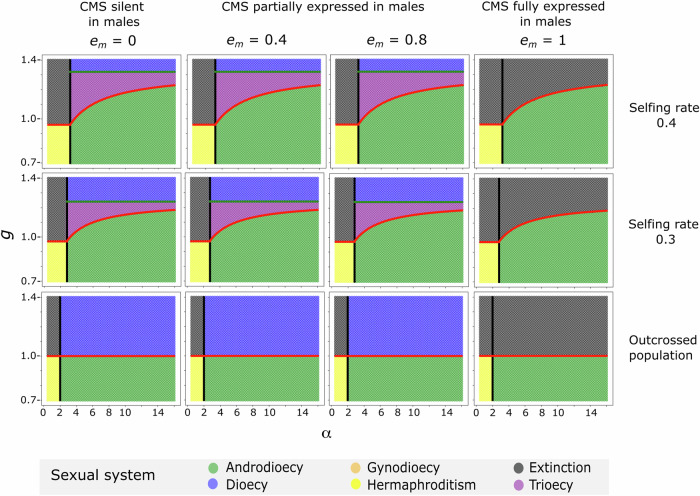
The sexual system at the equilibrium for populations without pollen limitation (Model 1), showing results for different selfing rates (rows) and the extent to which CMS is expressed in males (models in terms of *e*_m_ columns). In all panels, inbreeding depression *d* = 0.1. The lines in each panel are the thresholds for: males to invade a hermaphroditic population prior to CMS invasion (Eq. (1), black line); CMS invasion (Eqs. (2) or (3), red line); and CMS to be fixed after invading an androdioecious population (Eq. (5), green line). The sexual systems at equilibrium are denoted by the colours, defined in the inset legend. *g* and *α* refer to the relative seed production or pollen production, respectively, of females and males compared to that of hermaphrodites.

**Fig. 2:**
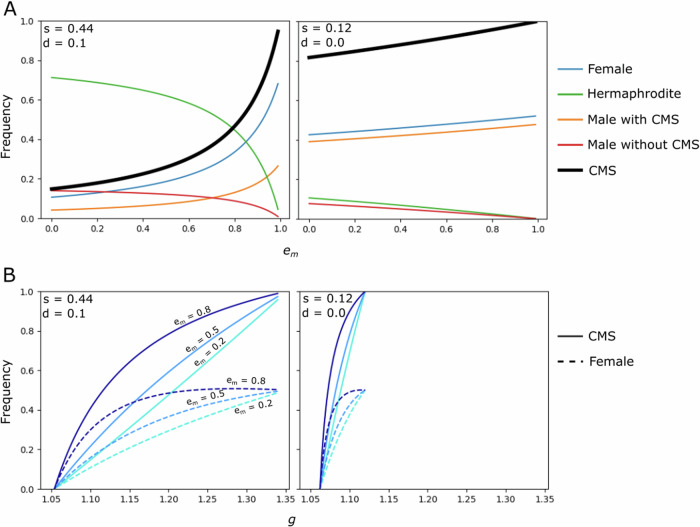
The frequencies of sexual morphs at equilibrium from model 1. The frequencies of the CMS cytotype, females, hermaphrodites, and males with and without the CMS cytotype at the trioecious equilibrium in model 1, as a function of *e*_m_, i.e., the effect of CMS on males (**A**) and of *g*, i.e., the relative seed production of females compared to that of hermaphrodites (**B**). *α* = 5 in all panels, *g* = 1.1, and *e*_m_ is plotted from 0 to 0.99 in panels (**A**). Note that the analytical solutions of frequencies of genotypes at trioecious equilibrium only applied to *e*_m_ approaches 1.0, i.e., where females still sire viable sons, which increases the fitness of males and causes the negative frequency depence selection on females and maintains stable trioecy. Values for *s* and *d* are given in the inset legends. The equations for the curves are given in Supplementary Information A.1.2.2.

**Fig. 3:**
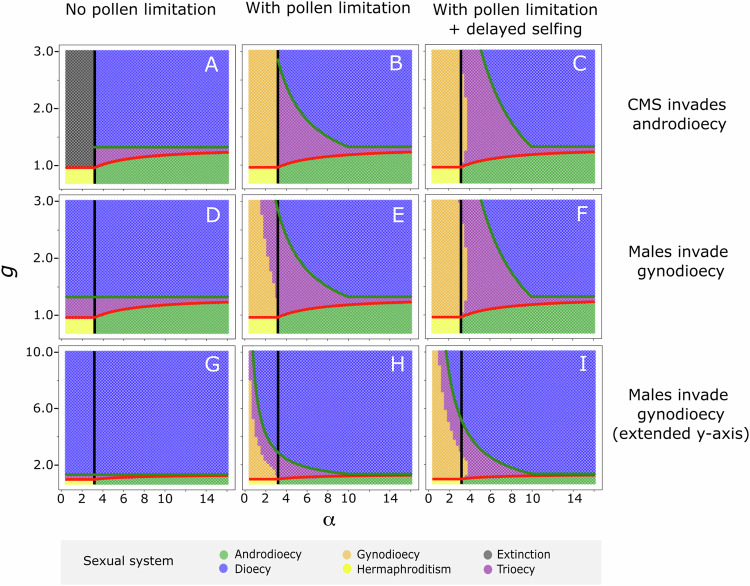
Sexual systems at equilibrium under three scenarios of pollen limitation (columns) and whether males or females are first to invade (rows). Left column, panels **A**, **D**, **G**: no pollen limitation (Model 1). Middle column, panels **B**, **E**, **H**: pollen limitation, such that ovules not fertilized by prior self or outcross pollen are aborted (Model 2). Right column, panels **C**, **F**, **I**: pollen limitation, such that hermaphrodites’ ovules that are not fertilized by prior self or outcross pollen are fertilized by an additional component of (delayed) selfing (Model 3). Upper row, panels **A**–**C**: scenarios in which CMS invades a hermaphroditic population or an androdioecious population at equilibrium. Middle and lower rows, panels **D**–**I**: scenarios in which males are introduced into a population already segregating for CMS (and prior to its eventual fixation for those cases where CMS would ultimately fix in the absence of male invasion). The lower row depicts the same scenario as the middle row with an extended y-axis. In all panels, *s* = 0.4, *d* = 0.1, and *e*_m_ = 0.8. The lines in each panel are the thresholds for: males to invade a hermaphroditic population prior to CMS invasion (Eq. (1), black line); CMS invasion (Eqs. (2) or (3), red line); CMS to be fixed after invading an androdioecious population (left column: Eq. (4); middle column: Eqs. (4) and (5), combined as Equation A8 in the Supplementary Information A.2; right column: Eqs. (4) and (6), combined as Equation A9 in Supplementary Information A.2; green line). The sexual systems at equilibrium are denoted by the colours, defined in the inset legend. *g* and *α* refer to the relative seed production or pollen production, respectively, of females and males compared to that of hermaphrodites.

Solution of the recursion equations confirms the conditions previously derived for the invasion of males into hermaphroditic populations (i.e., the evolution of androdioecy, Lloyd 1975; Charlesworth and Charlesworth 1978), as well as for the invasion of a CMS cytotype into a hermaphroditic population (Lewis 1941; Lloyd 1974a, 1974b, 1975). In the absence of pollen limitation, conditions for the invasion of CMS are identical to those for its fixation (and thus for theoretical population extinction), so that, in the absence of males, CMS cannot be maintained. However, in the presence of males that are not rendered completely sterile by CMS (i.e., as long as the Y chromosome can at least partially restore male fertility), the conditions for the stable maintenance of trioecy following the invasion of females exist. Interestingly, when hermaphrodites are partially self-fertilizing, the presence of males hinders both the invasion and the fixation of CMS. Finally, if males are rendered completely sterile by CMS, the invasion of CMS leads to its fixation (and theoretical population extinction). These results for populations in which female seed production is not pollen-limited (Model 1), are summarised in Figs. 1, 2. When seed production is pollen-limited (Models 2 and 3), conditions for the fixation of CMS are more restricted and trioecy is more frequently maintained (Fig. 3).

### Invasion of males and CMS into a hermaphroditic population

Solution of our recursion equations yields the expressions

1 $$\alpha \,>\, \frac{2(1-sd)}{1-s}$$

and

2 $$g \,>\, 1-sd$$

for the threshold fitness of males (*α*) and females (*g*), respectively, relative to hermaphrodites for the invasion of males and females into a hermaphroditic population. Thus, to invade an outcrossing population (*s* = 0), males need to be at least twice as successful as hermaphrodites at siring offspring, whereas the invasion of females requires only that their seed production exceeds that of hermaphrodites. In the absence of inbreeding depression, selfing has no effect on the invasion criterion for females, whereas selfing increasingly prevents males from invading (see also Fig. 1). These well-known results from previous analyses (Lloyd 1975; Charlesworth and Charlesworth 1978) have been invoked to explain both the high frequency of CMS in plants (Kaul 1988; Carlsson et al. 2008) as well as the rarity of androdioecy (Charlesworth 1984; Pannell 2002), because males must both produce much more pollen than hermaphrodites and will find it especially difficult to invade a partially selfing population (Charlesworth and Charlesworth 1978).

### Invasion of CMS into an androdioecious population

A key result is that the conditions for the invasion of CMS into a population Eq. (2) are modified by the presence of males, such that

3 $$g \,>\, 1-s+\frac{s(1-d)}{{P}_{{\rm{X}}}^{0}},$$

where

$${P}_{{\rm{X}}}^{0}=\frac{{f}_{{\rm{XX}},{\rm{n}}}^{0}+0.5\,\alpha {f}_{{\rm{XY}},{\rm{n}}}^{0}}{{f}_{{\rm{XX}},{\rm{n}}}^{0}+\alpha {f}_{{\rm{XY}},{\rm{n}}}^{0}}$$

is the fraction of pollen grains at the androdioecious equilibrium that do not have the Y chromosome (i.e., all pollen from hermaphrodites and half the pollen of males), and *f*^0^_XX,n_ and *f*^0^_XY,n_ are the frequencies of hermaphrodites and males, respectively. Equivalently,

$${P}_{{\rm{X}}}^{0}=\frac{1-(0.5\,\alpha -1){f}_{{\rm{XY}},{\rm{n}}}^{0}}{1-(\alpha -1){f}_{{\rm{XY}},{\rm{n}}}^{0}}.$$

Substitution into Eq. 3 in Charlesworth and Charlesworth (1978) for the frequency of males at an androdioecious equilibrium, i.e.,

$${f}_{{\rm{XY}},{\rm{n}}}^{0}=\frac{\alpha (1-s)-2(1-sd)}{2(\alpha -1)(1-sd)},$$

yields the criterion for CMS invasion in terms of the selfing rate, *s*, inbreeding depression, *d*, and the relative pollen production of males compared with hermaphrodites, *α* (see Supplementary Information A.2, Eq. A6). Because 0.5 $\le$ *P*^0^_X_ $\le$ 1.0 (as half the pollen produced by males carries a Y chromosome with an effective female-sterility mutation, so that *P*_X_^0^ is diminished by an increased frequency of males), Eq. (3) indicates that the presence of males hinders female invasion via CMS. This is because males do not transmit the CMS cytotype (because they do not produce seeds), and they therefore act as a CMS sink. Accordingly, the more pollen produced by males (the smaller the value of *P*^0^_X_), the more sons are produced by females and the greater the effect of the CMS sink in hindering female invasion and spread. These results are illustrated graphically in Fig. 1 for a range of different parameter values.

### Conditions for the fixation of CMS

In the absence of pollen limitation (see below), the conditions for invasion of CMS into a population are identical to those for its fixation when males are absent. In contrast, while the conditions for CMS invasion into an androdioecious population are given by Eq. (3), the conditions for its fixation in the presence of males are more stringent, with the requirement that

4 $$g\ge 1+s-2sd,$$

which corresponds to the invasion conditions in Eq. (3) with *P*^0^_X_ = 0.5, i.e., when males are at a frequency of 0.5 and the frequency of hermaphrodites approaches zero. Note that, barring one exception at *e*_m_ = 1 (males carrying CMS are fully sterile), the extent to which the CMS cytotype affects pollen production by males does not influence conditions for its invasion and fixation (*e*_m_ does not appear in Eqs. (3) and (4)). This is simply because, at the point of CMS invasion, males are not yet affected by male sterility and because, at the point of its fixation, *P*^0^_X_ = 0.5 regardless of the value of *e*_m_. When *e*_m_ = 1, conditions for CMS invasion and fixation are identical (Eq. (3); see also below). The difference between conditions for the invasion and those for the fixation of CMS corresponds to the parameter space in which trioecy can be maintained. Note that, although the value of *e*_m_ does not affect conditions for the invasion of CMS or its fixation, it does affect the dynamics and frequency of CMS in a trioecious population.

### Maintenance of trioecy

It is evident that trioecy in our model can only be maintained if both the CMS cytotype and males are maintained by negative frequency-dependent selection at an intermediate frequency. Previous models of the evolution of gynodioecy due to CMS have shown that the CMS cytotype can only be maintained at equilibrium if the hermaphrodites are partially selfing and female seed production is pollen-limited (Lewis 1941; Lloyd 1974a, 1974b, 1975), or if the population is also segregating for a costly fertility restorer allele, irrespective of whether seed set is pollen-limited (Charlesworth 1981; Delannay et al. 1981). In an extension to these previous results, our model indicates that CMS can be maintained in the absence of pollen limitation under some circumstances when males are also present, as long as the male determiner (e.g., a Y chromosome) carries the fertility restorer. Thus, the presence of males in a population that are at least partially immune to the effects of CMS establishes a condition for the maintenance of trioecy in which females are the result of CMS.

Trioecy is maintained by negative frequency-dependent selection acting on all three phenotypes. The presence of females increases the fitness of males by allowing the males to sire more ovules and therefore to produce more sons. In turn, the presence of males causes females to produce more sons that are incapable of transmitting the CMS cytotype. Together, these two effects establish conditions for the maintenance of both males and females with hermaphrodites: an increase in the frequency of CMS leads to an increase in the frequency of males, which however in turn hinders the further spread of CMS. Hermaphrodites should also benefit from the presence of females in the population through increased outcross siring opportunities. However, because the outcross siring success of hermaphrodites will typically be lower than that of males (as they produce less pollen), the maintenance of hermaphrodites can only be assured if they sire their own seeds by self-fertilization. Moreover, selfing by hermaphrodites protects them from producing sons sired by males, thereby increasing the fitness of the non-CMS cytotype and maintaining the CMS polymorphism and thus trioecy. Hence, to maintain all three phenotypes in the population, hermaphrodites need to self-fertilize (to protect the male fertile cytotype and increase its relative fitness when male frequency increases), and female spread needs to increase the males’ fitness i.e., females’ sons need to be partially restored and *e*_m_ < 1.

### Phenotype and genotype frequencies at equilibrium

The frequencies at which the different phenotypes and genotypes are maintained at a trioecious equilibrium depend on the value of all the parameters, i.e., the relative pollen and seed production of the different genotypes, the selfing rate of the hermaphrodites, the inbreeding depression suffered by selfed progeny, and the effect of the restorer allele on the Y chromosome (or, equivalently, the effect of CMS on the fertility of males). It is possible to derive an expression for these complex relations (Supplementary Information A.1.2.2), but little insight is gained.

Figure 2 presents examples of equilibrium frequencies for some representative parameter combinations. Two points are worth emphasising. First, the equilibrium frequencies of CMS and females can range from 0 to 1, but males are always maintained at frequencies <0.5. These results follow directly from the fact that CMS is transmitted to all ovules produced by seed-producing plants carrying it, whereas the male-determining Y is transmitted to only half of the progeny sired by males. And second, although the effect of CMS on males (*e*_m_) does not influence conditions for CMS invasion and fixation, the value of *e*_m_ does affect the CMS frequency in a trioecious population when all three sexual phenotype are present. More specifically, the higher the effect of CMS on males (i.e., the weaker the effect of fertility-restoring capacity of the Y), the higher the CMS frequency at the trioecious equilibrium. This is because a reduction in pollen produced by males (due to higher *e*_m_) reduces the proportion of sons (always sired by males) among the progeny of females, and this increases the proportion of the females’ daughters (sired by males and hermaphrodites), which transmit the CMS. Sons act as a CMS sink, because they produce no seeds.

### The effect of pollen limitation on the maintenance of trioecy

When the spread of females causes pollen limitation (by displacing pollen-producing individuals; Models 2 and 3), the CMS cytotype is ultimately prevented from fixing under wider conditions than when seed set is not pollen-limited (Fig. 3). This is because the fitness of females declines with their frequency due to the lack of pollen available to fertilize their ovules. Because this negative effect of pollen limitation on female fitness increases from zero when females are rare, the assumption that females cause pollen limitation has no bearing on female invasion itself. The effect of pollen limitation on the frequency of CMS and females is of course magnified when CMS is expressed in males (because this further reduces the amount of pollen in the population).

The threshold for CMS fixation in a model with pollen limitation is derived in Supplementary Information A.2. In the absence of pollen limitation, CMS will fix in a population in which females produce at least 1 – *s* + 2 *s*(1 – *d*) seeds compared with hermaphrodites, i.e., when females transmit more alleles than hermaphrodites to the next generation. However, with pollen limitation in a scenario in which all unfertilized ovules fail to develop into seeds (Model 2), trioecy will be maintained if

5 $$g \,<\, 1-s+\frac{4s(1-d)}{\alpha (1-{e}_{m})}.$$

Equation (5) clarifies the effect of the pollen production by males in the population, both in terms of reducing pollen limitation that compromises the seed production of females (because males produce *α* times more pollen relative to hermaphrodites), and in terms of the extent to which their pollen production is reduced by expression of the CMS cytotype.

When ovules produced by hermaphrodites that are not outcrossed (because of pollen limitation) can be fertilized by delayed selfing (Model 3), trioecy is maintained under broader conditions than when these ovules remain unfertilized and abort (Model 2), because delayed selfing increases the fitness of hermaphrodites and hinders the fixation of CMS and thus prevents a transition from trioecy to dioecy. Specifically, under these conditions of pollen limitation, CMS is prevented from fixation (and trioecy may be maintained) if

6 $$g \,<\, (1-s)(2d-1)+\frac{4(1-d)}{\alpha (1-{e}_{m})}.$$

Importantly, Eqs. (5) and (6) will only apply when the average pollen production per individual in a dioecious population, *α*(1 *– e*_m_)/2, is smaller than the threshold below which pollen limitation occurs. This clarifies why Wolf and Takebayashi (2004) found no conditions for the maintenance of trioecy in their model with delayed self-fertilization: their model assumes the dioecious population are not pollen-limited. Note that, in Model 3, the selfing rate necessarily increases with pollen limitation, reducing the prospective fitness of males. The invasion of CMS into an androdioecious population thus leads to gynodioecy via a transitory trioecious intermediate state. As a consequence, the parameter space for the maintenance of gynodioecy is slightly expanded (Fig. 3C). This result concurs with that of the equivalent delayed self-fertilization model of Wolf and Takebayashi (2004).

### Effect of the order of the invasion of unisexual phenotypes on the sexual system

As in any situation in which different phenotypes are maintained at equilibrium by negative frequency-dependent selection, whether males or females first evolve in the sequence of steps towards trioecy has no effect on their frequencies at equilibrium, except for the case where one phenotype is excluded by the fixation of a second before the third arrives (Fig. 3). In particular, if CMS invades a hermaphroditic population in which there is no pollen limitation, then the population is expected to become extinct unless males invade beforehand (left-hand-side region of the black line in Fig. 3A, D, G). In contrast, if males are able to first invade a hermaphroditic population, an initial androdioecious equilibrium will be reached, and the subsequent invasion of CMS will lead to the evolution of either dioecy or trioecy, but not extinction (unless CMS is fully expressed in males, too; see above) (Figs. 1, 3). In a scenario in which seed production is pollen-limited, there is a combination of parameter values that allow the maintenance of trioecy when CMS invades before males (Fig. 3E, F, H, I), whereas gynodioecy is maintained in this parameter space if males are not available to invade prior to CMS invasion (compare the region left of the black line in Fig. 3B, C with Fig. 3E, F). This is simply because the additional parameter space that permits the maintenance of trioecy also corresponds to a situation in which males produce too little pollen to invade a hermaphroditic population, but males could invade a population already maintaining females. These details complement results from the models of Schultz (1994) and Maurice et al. (1994) which considered the evolution of dioecy via gynodioecy with CMS, though these models did not consider androdioecy as the first step.

## Discussion

Our model has considered conditions for the evolution and maintenance of cytoplasmic male sterility in populations also segregating for males and hermaphrodites. It thus advances previous theoretical research by investigating the evolution and maintenance of trioecy involving cytoplasmic (rather than nuclear) male sterility and by determining how the presence of males, and thus partial separation of the sexes, affects the fate of cytoplasmic male sterility. Our results are also relevant to models for transitions between combined and separate sexes, as trioecy must often be an intermediate step in the evolution of dioecy via the spread of male and female sterility mutations, with male sterility potentially initially caused by cytoplasmic factors. We discuss these points briefly below.

Previous analyses of a model for the evolution of trioecy (Maurice and Fleming 1995) have pointed to the necessity of pollen limitation in combination with partial self-fertilization by hermaphrodites for the maintenance of all three sexual phenotypes at intermediate frequencies in the population. Our model confirms the necessity of partial self-fertilization by hermaphrodites for the maintenance of trioecy but, further, indicates that pollen limitation is not necessary if females are caused by a cytoplasmic male-sterility mutation and males are present and not fully sterilized by the same mutation. This is because males can carry a CMS mutation but are at least partially protected from its sterilizing effects; they thus continue to transmit the Y chromosome (and the restorer) but not, of course, the CMS mutation, which is only transmitted through ovules. In this sense, males play the same role as autosomal restorers in canonical models for the maintenance of gynodioecy with nuclear-cytoplasmic gynodioecy (Charlesworth 1981; Delannay et al. 1981), with the difference that males not only express a male-fertility restorer but are also female-sterile. We note that although our model finds conditions for the maintenance of trioecy in the absence of pollen limitation, it also shows that pollen limitation expands the parameter space for the maintenance of trioecy.

Whereas the presence of males permits the maintenance of trioecy, interestingly they also make it more difficult for CMS to invade and to be maintained. This is because all sons produced by females will carry the CMS mutation but cannot transmit it, so that they act as a CMS sink. In a model that also assumed the fertility-restoring capacity of an effective Y chromosome, Schultz (1994) also found that the presence of CMS in a population promoted the invasion of males. Whereas Schultz (1994) focused his analysis on the invasion of males into a population segregating for CMS, our model considers the reverse situation, i.e., the invasion of CMS into an androdioecious population. Our results reveal a certain asymmetry in the way in which the presence of unisexual individuals affects the invasion of the opposite sex, namely because the presence of males hinders rather than promotes the invasion of the opposite sex (i.e., CMS females). This difference can be attributed to the different roles of the two sexes in such models in terms of whether they act as a transmission sink (males) or not (hermaphrodites or females).

Our model addresses a scenario that must be rare in nature and thus contributed to an understanding of this rarity. Not only is the focal point of departure, androdioecy, exceedingly rare in flowering plants (Charlesworth 1984; Pannell 2002), but, to our knowledge (barring one exception), none of the few cases of trioecy itself investigated to date have revealed females caused by CMS (Del et al. 1988; Fleming et al. 1994; Maurice and Fleming 1995; Silva et al. 2008; Del Castillo and Argueta 2009; Joseph and Murthy 2015; Oyarzún et al. 2020). The one potential exception is the case of hexaploid *Mercurialis annua*, an androdioecious species (Pannell 1997a, 1997d; Pannell et al. 2008) in which male sterility has been recorded (Perry et al. 2012) and appears to be caused by CMS (Nguyen et al. 2024).

The female frequencies of trioecious populations of *M. annua* tend always to be low and are never higher than about 40% (Nguyen et al. 2024). Our model found conditions under which female frequencies in trioecious populations could be maintained at 40% and lower, conditions which perhaps apply to the case of *M. annua*. For instance, seed set by females in populations of *M. annua* tend not to be pollen-limited, seed production by females is only slightly greater than by hermaphrodites, and the selfing rate of hermaphrodites tends to be low—conditions under which trioecy is stable and female frequencies are often predicted to be low in our model. Nguyen et al. (2024) presented a detailed analysis of phenotypes and fitness components for natural trioecious populations of *M. annua*. Our assumption that the Y chromosome is linked to a restorer is also supported by data from crosses of *M. annua* in which females produced fertile males (Nguyen et al. 2024). The evolution of this genetic architecture remains to be elucidated, but verbal hypotheses were advanced by Schultz (1994) that invoke selection for a restorer when CMS reaches a high frequency and selection favouring males with a Y chromosome linked to the restorer preventing the production of neuter individuals expressing both male and female sterility.

Previous work indicates that the androdioecious populations of *M. annua* into which CMS has evidently invaded are part of a metapopulation in which hermaphrodites enjoy an advantage over males of reproductive assurance via selfing during the colonization of new populations, whereas males enjoy high siring success as migrants into established populations (Pannell 1997c, 2000; Pannell et al. 2014). Our model here is deterministic and so does not consider the effects of demographic stochasticity in small populations, nor the effects of selection against females in recently colonized populations lacking mates (McCauley and Taylor 1997; Pannell 1997b). Previous models have considered the dynamics and evolution of CMS in a gynodioecious metapopulations (Lewis 1941; Lloyd 1974a, 1974b, 1975; Charlesworth and Ganders 1979; Charlesworth 1981; Delannay et al. 1981; Ross and Gregorius 1985; Frank 1989; Gouyon et al. 1991; Couvet et al. 1998; Frank and Barr 2001; Dufaÿ et al. 2007; Dufay and Pannell 2010), but none, to our knowledge, has yet considered the evolution of trioecy in metapopulations with extinction and colonisation dynamics. Such a model would likely throw further light on the occurrence and maintenance of trioecy, not least on the uniformly low frequency of females in *M. annua*, in particular, and on its rarity in plants more generally.

The results of our model are also relevant more generally to evolutionary paths between combined and separate sexes. Canonical models for the evolution of dioecy from hermaphroditism implicitly invoke a trioecious intermediate stage, e.g., when males caused by female-sterility mutation transmitted in the nucleus invade and spread in a gynodioecious population (Charlesworth and Charlesworth 1978; Bawa 1980). The evolution of dioecy via androdioecy has also been considered, though more rarely (because androdioecy itself is so rare; Charlesworth 1984; Pannell 2002). There have been numerous models of the evolution of CMS in hermaphroditic populations (see Introduction), and Schultz (1994) investigated the potential role of CMS in the evolution of dioecy, whereby the determination of male sterility passes from the cytoplasmic factor (which is maternally inherited and thus cannot determine sex in a fully dioecious population) to the biparentally inherited fertility restorer factor when CMS is driven to fixation. Our model advances on Schultz’s (1994) analysis by considering androdioecy as a first step and by focusing attention on the maintenance and the potential stability (or transience) of the potentially intermediate state of trioecy.

## Supplementary information

Supplementary Information

Output of the Mathematica script - Model 1

Output of the Mathematica script - Model 2

Output of the Mathematica script - Model 3

## Data Availability

The Mathematica scripts and the simulation codes (in Python) are deposited on https://github.com/maithunguyen/Trioecy-model-single-population.
