# Supplementary material for: The evolution and maintenance of trioecy with cytoplasmic male sterility
Source: Heredity (Edinb). 2024 Oct 14;134(1):1–9. doi: 10.1038/s41437-024-00729-7 (PMC11723941; doi:10.1038/s41437-024-00729-7)
Supplement: Supplementary file 1 — Supplementary Information [file 41437_2024_729_MOESM1_ESM.pdf]

A Supplementary Information

“The evolution and maintenance of trioecy with cytoplasmic male sterility”

M. T. Nguyen* and J. R. Pannell

Department of Ecology and Evolution
University of Lausanne
1015 Lausanne
Switzerland

Contents

A.1 The invasion of a CMS mutation without considering pollen limitation (Model 1) . . . 2
A.1.1 Invasion of a CMS mutation into a hermaphroditic population . . . 2
A.1.2 The invasion of CMS into an androdioecious population . 3
A.1.2.1 Thresholds for CMS invasion and fixation 3
A.1.2.2 Calculation of genotype frequencies at equilibrium 5
A.2 The effect of pollen limitation (Models 2 and 3) . . . 7
A.2.1 Pollen limitation with leftover hermaphroditic ovules aborted (Model 2) . . . 8
A.2.2 Pollen limitation where leftover hermaphroditic ovules can be reused for self-fertilization (Model 3) . . . 9

A.1 The invasion of a CMS mutation without considering pollen limitation (Model 1)

Let Y be a dominant nuclear female-sterility mutation. Let c be the cytotype carrying the male-sterility mutation and n be the male-fertile cytotype. The frequency of each genotype is denoted as follows:

- $f^t_{\mathrm{XX,n}}$: frequency of hermaphrodites at generation t,
- $f^t_{\mathrm{XY,n}}$: frequency of males at generation t,
- $f^t_{\mathrm{XX,c}}$: frequency of females at generation t,
- $f^t_{\mathrm{XY,c}}$: frequency of males carrying the CMS mutation at generation t.

A.1.1 Invasion of a CMS mutation into a hermaphroditic population

In the case of females invading a hermaphroditic population, we only need to account for the frequency of these two genotypes. We have

$$f^t_{\mathrm{XX,n}} + f^t_{\mathrm{XX,c}} = 1.$$

The change in the female frequency at the beginning determines the fate of the population. Let $r^t_{\mathrm{XX,c}}$ and $r^t_{\mathrm{XX,n}}$ be the growth rate of the female and hermaphrodite frequencies in the population at time t, respectively. We then have $r^t_{\mathrm{XX,c}} = f^{t+1}_{\mathrm{XX,c}}/f^t_{\mathrm{XX,c}} - 1$ and $r^t_{\mathrm{XX,n}} = f^{t+1}_{\mathrm{XX,n}}/f^t_{\mathrm{XX,n}} - 1$.

From the recurrence equation for the female frequency, we can calculate its growth rate $r^t_{\mathrm{XX,c}}$ as follows:

$$\begin{aligned}
f^{t+1}_{\mathrm{XX,c}} &= \frac{f^t_{\mathrm{XX,c}} g}{f^t_{\mathrm{XX,n}}(1-s) + f^t_{\mathrm{XX,n}} s(1-d) + f^t_{\mathrm{XX,c}} g} \\
&= \frac{f^t_{\mathrm{XX,c}} g}{f^t_{\mathrm{XX,n}}(1-s+s-sd) + f^t_{\mathrm{XX,c}} g} \\
&= \frac{f^t_{\mathrm{XX,c}} g}{(1-f^t_{\mathrm{XX,c}})(1-sd) + f^t_{\mathrm{XX,c}} g} \\
\Leftrightarrow \frac{f^{t+1}_{\mathrm{XX,c}} - f^t_{\mathrm{XX,c}}}{f^t_{\mathrm{XX,c}}} &= \frac{g}{(1-f^t_{\mathrm{XX,c}})(1-sd) + f^t_{\mathrm{XX,c}} g} - 1 \\
\Leftrightarrow r^t_{\mathrm{XX,c}} &= \frac{g}{(1-f^t_{\mathrm{XX,c}})(1-sd) + f^t_{\mathrm{XX,c}} g} - 1.
\end{aligned}$$

Whether CMS can invade and be fixed is determined by the growth rate for the female frequency at time $t = 0$, i.e., $r^0_{\mathrm{XX,c}}$. If $r^0_{\mathrm{XX,c}} > 0$, CMS will invade and spread. We can further elaborate the lower bound threshold of CMS invasion

$r^0_{\mathrm{XX,c}} = 0$ as follows:

$$\begin{aligned} 0 &= r^0_{\mathrm{XX,c}} \\ \Leftrightarrow 1 &= \frac{g}{(1 - f^0_{\mathrm{XX,c}})(1 - sd) + f^0_{\mathrm{XX,c}} g} \\ \Leftrightarrow g &= (1 - f^0_{\mathrm{XX,c}})(1 - sd) + f^0_{\mathrm{XX,c}} g \\ \Leftrightarrow g(1 - f^0_{\mathrm{XX,c}}) &= (1 - f^0_{\mathrm{XX,c}})(1 - sd) \\ \Leftrightarrow g &= 1 - sd. \end{aligned} \tag{A1}$$

A.1.2 The invasion of CMS into an androdioecious population

For androdioecy to evolve, males need to produce more pollen than a certain threshold (Charlesworth and Charlesworth, 1978):

$$\alpha = \frac{2(1 - sd)}{1 - s}. \tag{A2}$$

A.1.2.1 Thresholds for CMS invasion and fixation

Let P^t_{X} be the fraction of pollen not carrying the female-sterility mutation at generation t. Let $r^t_{\mathrm{XX,c}}$ and $r^t_{\mathrm{XX,n}}$ be the growth rate of the female and hermaphrodite frequencies in the population at time t, respectively, as defined in section A.1.1. At any time point after CMS invasion, we have

$$\begin{aligned} f^{t+1}_{\mathrm{XX,n}} &= \frac{f^t_{\mathrm{XX,n}}(1 - s)P^t_{\mathrm{X}} + f^t_{\mathrm{XX,n}} s(1 - d)}{f^t_{\mathrm{XX,n}}(1 - sd) + f^t_{\mathrm{XX,c}} g} \\ f^{t+1}_{\mathrm{XX,c}} &= \frac{f^t_{\mathrm{XX,c}} P^t_{\mathrm{X}} g}{f^t_{\mathrm{XX,n}}(1 - sd) + f^t_{\mathrm{XX,c}} g} \\ \Rightarrow \frac{f^{t+1}_{\mathrm{XX,c}}}{f^{t+1}_{\mathrm{XX,n}}} &= \frac{f^t_{\mathrm{XX,c}} P^t_{\mathrm{X}} g}{f^t_{\mathrm{XX,n}}(1 - s)P^t_{\mathrm{X}} + f^t_{\mathrm{XX,n}} s(1 - d)} \\ \Leftrightarrow \frac{f^{t+1}_{\mathrm{XX,c}} \div f^t_{\mathrm{XX,c}}}{f^{t+1}_{\mathrm{XX,n}} \div f^t_{\mathrm{XX,n}}} &= \frac{P^t_{\mathrm{X}} g}{(1 - s)P^t_{\mathrm{X}} + s(1 - d)} \\ \Leftrightarrow \frac{r^t_{\mathrm{XX,c}} + 1}{r^t_{\mathrm{XX,n}} + 1} &= \frac{P^t_{\mathrm{X}} g}{(1 - s)P^t_{\mathrm{X}} + s(1 - d)} \\ \Leftrightarrow \frac{r^t_{\mathrm{XX,c}} - r^t_{\mathrm{XX,n}}}{r^t_{\mathrm{XX,n}} + 1} &= \frac{g}{1 - s + \frac{s(1-d)}{P^t_{\mathrm{X}}}} - 1. \end{aligned} \tag{A3}$$

Note that the growth rate of the hermaphrodite frequency is $r^t_{\mathrm{XX,n}} > -1$ (as defined in A.1.1). The denominator on the left side of equation (A3) is always positive. Whether the difference in the growth rate of the female and hermaphrodite frequencies are positive or negative, therefore, only depends on the sign of the right side of equation (A3). More precisely, $r^t_{\mathrm{XX,c}} - r^t_{\mathrm{XX,n}} \geq 0$ when

$$\frac{g}{1-s+\frac{s(1-d)}{P^t_{\mathrm{X}}}} > 1. \tag{A4}$$

For $s(1-d) > 0$, the left side of inequality (A4) is monotonically increasing over P^t_{X}. The CMS invades when inequality (A4) is satisfied with the specific value of P^t_{X} at the moment of invasion. For $P^t_{\mathrm{X}} = 1$, i.e., the value at the moment of CMS invasion into a hermaphroditic population, inequality (A4) is then $g \geq 1 - sd$. The minimum female fitness g required for the CMS to successfully invade is $g = 1 - sd$, identical to equation (A1). For $P^t_{\mathrm{X}} = 0.5$, i.e., the value at the moment of CMS fixation, reformulating inequality (A4) gives us the threshold of g for CMS fixation:

$$\begin{aligned}
1 &= \frac{0.5g}{(1-s)0.5 + s(1-d)} \\
\Leftrightarrow 0.5g &= (1-s)0.5 + s(1-d) \\
\Leftrightarrow g &= (1-s) + 2s(1-d) \\
\Leftrightarrow g &= 1 + s - 2sd.
\end{aligned} \tag{A5}$$

At the beginning of the invasion, P_{X} is the largest, because once females increases the frequency, the male frequency will also increase, so as the proportion of P_{Y}, which is $1 - P_{\mathrm{X}}$ (see main text for more detail of the frequencies dynamics). This largest P_{X} is, therefore, also the fraction of pollen not carrying the female-sterility mutation at the equilibrium of an androdioecious population. Let $f^0_{\mathrm{XX,n}}$ and $f^0_{\mathrm{XY,n}}$ be the hermaphrodite and male frequencies, respectively, in an androdioecious population at the equilibrium. At time point 0, we have

$$\begin{aligned}
P^0_{\mathrm{X}} &= \frac{f^0_{\mathrm{XX,n}} + f^0_{\mathrm{XY,n}}\alpha 0.5}{f^0_{\mathrm{XX,n}} + f^0_{\mathrm{XY,n}}\alpha} \\
&= \frac{1 - f^0_{\mathrm{XY,n}}(0.5\alpha - 1)}{1 - f^0_{\mathrm{XY,n}}(\alpha - 1)}.
\end{aligned}$$

As shown in previous work (Charlesworth and Charlesworth, 1978), the frequency of males at the equilibrium of the androdioecious population is

$$f^0_{\mathrm{XY,n}} = \frac{\alpha(1-s) - 2(1-sd)}{2(\alpha-1)(1-sd)}.$$

The threshold for g that allows CMS to invade an androdioecious population is

$$\begin{aligned}
1 &= \frac{g}{1-s+\frac{s(1-d)}{P^0_{\mathrm{X}}}} \\
\Leftrightarrow g &= 1 - s + \frac{s(1-d)}{P^0_{\mathrm{X}}} \\
&= 1 - s + \frac{s(1-d)\left(1 - \frac{\alpha(1-s)-2(1-sd)}{2(\alpha-1)(1-sd)}(\alpha-1)\right)}{1 - \frac{\alpha(1-s)-2(1-sd)}{2(\alpha-1)(1-sd)}(0.5\alpha - 1)}.
\end{aligned} \tag{A6}$$

A.1.2.2 Calculation of genotype frequencies at equilibrium

Let the superscript $*$ denotes the value of the variable at equilibrium. At equilibrium, the left side of inequality (A4) = 1. From this, we can calculate P_{X}^*, as well as the frequency of pollen carrying the female-sterility mutation, P_{Y}^*, at equilibrium:

$$P_{\mathrm{X}}^* = \frac{s(1-d)}{g+s-1},$$
$$P_{\mathrm{Y}}^* = 1 - P_{\mathrm{X}}^* = \frac{g+sd-1}{g+s-1}.$$

Let $f_{\mathrm{XY,n}}^*$, $f_{\mathrm{XX,n}}^*$, $f_{\mathrm{XY,c}}^*$ and $f_{\mathrm{XX,c}}^*$ be the frequencies of males, hermaphrodites, males carrying the CMS, and females, respectively, at equilibrium. Let e_{m} be the expression rate of CMS in males carrying them. We can also calculate P_{Y}^* from the pollen production of each phenotype in the population:

$$P_{\mathrm{Y}}^* = \frac{0.5\alpha(f_{\mathrm{XY,n}}^* + f_{\mathrm{XY,c}}^*(1-e_{\mathrm{m}}))}{f_{\mathrm{XX,n}}^* + \alpha(f_{\mathrm{XY,n}}^* + f_{\mathrm{XY,c}}^*(1-e_{\mathrm{m}}))}.$$

Let N_S^* be the total number of seeds in the population:

$$N_S^* = f_{\mathrm{XX,n}}^*(1-sd) + f_{\mathrm{XX,c}}^* g.$$

We further have the recurrence equations evaluated at equilibrium:

$$f_{\mathrm{XX,n}}^* = \frac{f_{\mathrm{XX,n}}^*(1-s)P_{\mathrm{X}}^* + f_{\mathrm{XX,n}}^* s(1-d)}{N_S^*},$$
$$f_{\mathrm{XY,n}}^* = \frac{f_{\mathrm{XX,n}}^*(1-s)P_{\mathrm{Y}}^*}{N_S^*},$$
$$f_{\mathrm{XX,c}}^* = \frac{f_{\mathrm{XX,c}}^* g P_{\mathrm{X}}^*}{N_S^*},$$
$$f_{\mathrm{XY,c}}^* = \frac{f_{\mathrm{XX,c}}^* g P_{\mathrm{Y}}^*}{N_S^*}. \tag{A7}$$

At equilibrium, the frequency of each phenotype is constant. From the equation

for the female frequency we have

$$
\begin{aligned}
N_S^* &= gP_{\mathrm{X}}^* \\
\Leftrightarrow f_{\mathrm{XX,n}}^*(1-sd) + f_{\mathrm{XX,c}}^* g &= gP_{\mathrm{X}}^* \\
\Leftrightarrow f_{\mathrm{XX,c}}^* &= \frac{gP_{\mathrm{X}}^* - f_{\mathrm{XX,n}}^*(1-sd)}{g} \\
&= P_{\mathrm{X}}^* - \frac{f_{\mathrm{XX,n}}^*(1-sd)}{g} \\
\Leftrightarrow f_{\mathrm{XY,c}}^* &= (P_{\mathrm{X}}^* - \frac{f_{\mathrm{XX,n}}^*(1-sd)}{g}) * \frac{P_{\mathrm{Y}}^*}{P_{\mathrm{X}}^*} \\
&= P_{\mathrm{Y}}^* - \frac{f_{\mathrm{XX,n}}^*(1-sd)(P_{\mathrm{Y}}^*)}{gP_{\mathrm{X}}^*} \\
\Leftrightarrow f_{\mathrm{XY,n}}^* &= \frac{f_{\mathrm{XX,n}}^*(1-s)P_{\mathrm{Y}}^*}{gP_{\mathrm{X}}^*} \\
\Leftrightarrow f_{\mathrm{XY,n}}^* + f_{\mathrm{XY,c}}^*(1-e_{\mathrm{m}}) &= \frac{f_{\mathrm{XX,n}}^*(1-s)P_{\mathrm{Y}}^*}{gP_{\mathrm{X}}^*} + (P_{\mathrm{Y}}^* - \frac{f_{\mathrm{XX,n}}^*(1-sd)(P_{\mathrm{Y}}^*)}{gP_{\mathrm{X}}^*})(1-e_{\mathrm{m}}) \\
&= P_{\mathrm{Y}}^*(\frac{f_{\mathrm{XX,n}}^*(1-s)}{gP_{\mathrm{X}}^*} + (1 - \frac{f_{\mathrm{XX,n}}^*(1-sd)}{gP_{\mathrm{X}}^*})(1-e_{\mathrm{m}})) \\
&= P_{\mathrm{Y}}^*(1-e_{\mathrm{m}} + f_{\mathrm{XX,n}}^*(\frac{1-s}{gP_{\mathrm{X}}^*} - \frac{(1-e_{\mathrm{m}})(1-sd)}{gP_{\mathrm{X}}^*})) \\
&= P_{\mathrm{Y}}^*(1-e_{\mathrm{m}} + f_{\mathrm{XX,n}}^*(\frac{1-s-(1-e_{\mathrm{m}})(1-sd)}{gP_{\mathrm{X}}^*})).
\end{aligned}
$$

On the other hand, we have

$$
\begin{aligned}
P^*_{\mathrm{Y}} &= \frac{0.5\alpha(f^*_{\mathrm{XY,n}} + f^*_{\mathrm{XY,c}}(1-e_{\mathrm{m}}))}{f^*_{\mathrm{XX,n}} + \alpha(f^*_{\mathrm{XY,n}} + f^*_{\mathrm{XY,c}}(1-e_{\mathrm{m}}))} \\
\Leftrightarrow 1 - 2P^*_{\mathrm{Y}} &= \frac{f^*_{\mathrm{XX,n}}}{f^*_{\mathrm{XX,n}} + \alpha(f^*_{\mathrm{XY,n}} + f^*_{\mathrm{XY,c}}(1-e_{\mathrm{m}}))} \\
\Leftrightarrow \frac{1}{1-2P^*_{\mathrm{Y}}} &= 1 + \frac{\alpha(f^*_{\mathrm{XY,n}} + f^*_{\mathrm{XY,c}}(1-e_{\mathrm{m}}))}{f^*_{\mathrm{XX,n}}} \\
\Leftrightarrow \frac{2P^*_{\mathrm{Y}} f^*_{\mathrm{XX,n}}}{1-2P^*_{\mathrm{Y}}} &= \alpha(f^*_{\mathrm{XY,n}} + f^*_{\mathrm{XY,c}}(1-e_{\mathrm{m}})) \\
\Leftrightarrow \frac{2P^*_{\mathrm{Y}} f^*_{\mathrm{XX,n}}}{1-2P^*_{\mathrm{Y}}} &= \alpha P^*_{\mathrm{Y}}(1 - e_{\mathrm{m}} + f^*_{\mathrm{XX,n}}(\frac{1-s-(1-e_{\mathrm{m}})(1-sd)}{gP^*_{\mathrm{X}}})) \\
\Leftrightarrow 2f^*_{\mathrm{XX,n}} &= \alpha(1-2P^*_{\mathrm{Y}})(1 - e_{\mathrm{m}} + f^*_{\mathrm{XX,n}}(\frac{1-s-(1-e_{\mathrm{m}})(1-sd)}{gP^*_{\mathrm{X}}})) \\
\Leftrightarrow \alpha(1-2P^*_{\mathrm{Y}})(1-e_{\mathrm{m}}) &= f^*_{\mathrm{XX,n}}(2 - \alpha(1-2P^*_{\mathrm{Y}})(\frac{1-s-(1-e_{\mathrm{m}})(1-sd)}{gP^*_{\mathrm{X}}})) \\
\Leftrightarrow f^*_{\mathrm{XX,n}} &= \frac{\alpha(1-2P^*_{\mathrm{Y}})(1-e_{\mathrm{m}})gP^*_{\mathrm{X}}}{2gP^*_{\mathrm{X}} - \alpha(1-2P^*_{\mathrm{Y}})(1-s-(1-e_{\mathrm{m}})(1-sd))} \\
\Leftrightarrow f^*_{\mathrm{XX,n}} &= \frac{\alpha(2P^*_{\mathrm{X}}-1)(1-e_{\mathrm{m}})gP^*_{\mathrm{X}}}{2gP^*_{\mathrm{X}} - \alpha(2P^*_{\mathrm{X}}-1)(1-s-(1-e_{\mathrm{m}})(1-sd))}.
\end{aligned}
$$

where $P^*_{\mathrm{X}} = \frac{s(1-d)}{g+s-1}$. $f^*_{\mathrm{XY,n}}, f^*_{\mathrm{XX,c}}$ and $f^*_{\mathrm{XY,c}}$ can be calculated from $f^*_{\mathrm{XX,n}}$ following the system of equations (A7). They depend only on α, s, d, g and e_{m} and not on the initial frequencies. The frequency of CMS is the sum of $f^*_{\mathrm{XX,c}}$ and $f^*_{\mathrm{XY,c}}$.

A.2 The effect of pollen limitation (Models 2 and 3)

We assume that pollen limitation occurs when the average pollen produced per individual (averaged over all hermaphrodites, males and females), is less than a threshold pollen amount P. Under pollen limitation, the proportion of fertilized outcrossed seeds decreases linearly from 1 to 0 with a decrease in average pollen production from P to 0. Let L^t and P^t be the proportion of outcrossed seeds that is fertilized, and the average pollen produced per individual, respectively, at time t. $L^t = min\{1, \frac{P^t}{P}\}$.

For simulations and further calculation, we assume that pollen limitation occurs when pollen production falls below that of a hermaphroditic population. The average pollen produced per individual of a hermaphroditic population is then $P = f^*_{\mathrm{XX,n}} \times 1 = 1$, where $f^*_{\mathrm{XX,n}} = 1$. The proportion of outcrossed seeds that is fertilized at time t is then $L^t = min\{1, \frac{P^t}{P}\} = min\{1, P^t\}$.

A.2.1 Pollen limitation with leftover hermaphroditic ovules aborted (Model 2)

Equation (A3), talking into account the effect of pollen limitation, is

$$\begin{aligned} r^t_{\mathrm{XX,c}} - r^t_{\mathrm{XX,n}} &\sim \frac{L^t g P^t_{\mathrm{X}}}{(1-s)L^t P^t_{\mathrm{X}} + s(1-d)} \\ \Leftrightarrow r^t_{\mathrm{XX,c}} - r^t_{\mathrm{XX,n}} &\sim \frac{g}{1-s+\frac{s(1-d)}{L^t P^t_{\mathrm{X}}}}. \end{aligned}$$

Analogous to the derivation and argument in section A.1.2.1, the condition for CMS fixation is then $g = 1 - s + \frac{s(1-d)}{L^t P^t_{\mathrm{X}}}$, with value L^t and P^t_{X} at the point of fixation, i.e., half of the population is comprised of males carrying CMS and the other half is comprised of females. The proportion of pollen not carrying the female-sterility mutation is then $P^t_{\mathrm{X}} = 0.5$, because, at the dioecious transition, all pollen producers are males, which gives $P^t_{\mathrm{X}} = P^t_{\mathrm{Y}} = 0.5$. The average pollen produced per individual at this point is $P^t = 0.5\alpha(1-e_{\mathrm{m}})$, where 0.5 is the frequency of males in the population and $\alpha(1-e_{\mathrm{m}})$ is the pollen production of a male carrying CMS.

At transition to dioecy, we have

$$\begin{aligned} L^t P^t_{\mathrm{X}} &= min\{1, P^t\} \times P^t_{\mathrm{X}} \\ \Leftrightarrow L^t P^t_{\mathrm{X}} &= min\{1, \alpha(1-e_{\mathrm{m}})0.5\}0.5 \\ &= min\{0.5, 0.25\alpha(1-e_{\mathrm{m}})\}. \end{aligned}$$

Inserting this expansion of $L^t P^t_{\mathrm{X}}$ into inequality (A4), the threshold for CMS fixation under possible pollen limitation is

$$\begin{aligned} g &= 1 - s + \frac{s(1-d)}{min\{0.5, 0.25\alpha(1-e_{\mathrm{m}})\}} \\ \Leftrightarrow g &= max\left\{1+s-2sd, 1-s+\frac{s(1-d)}{0.25\alpha(1-e_{\mathrm{m}})}\right\} \\ \Leftrightarrow g &= max\left\{1+s-2sd, 1-s+\frac{4s(1-d)}{\alpha(1-e_{\mathrm{m}})}\right\}, \end{aligned} \tag{A8}$$

where the left term of the right side is applied if there is more pollen in the population than the pollen limitation threshold, and the right term is applied when it is less. The threshold for CMS fixation under pollen limitation is then

$$g = 1 - s + \frac{4s(1-d)}{\alpha(1-e_{\mathrm{m}})}. \tag{A8.5}$$

For general cases where the pollen limitation threshold is an arbitrary value P instead of value 1, $L^t P^t_{\mathrm{X}} = min\{1, P^t/P\}P^t_{\mathrm{X}}$ and the equation (A8.5) becomes $g = 1 - s + \frac{4s(1-d)P}{\alpha(1-e_{\mathrm{m}})}$.

A.2.2 Pollen limitation where leftover hermaphroditic ovules can be reused for self-fertilization (Model 3)

Talking into account the effect of pollen limitation, equation (A3) becomes

$$r^t_{\mathrm{XX,c}} - r^t_{\mathrm{XX,n}} \sim \frac{LgP^t_{\mathrm{X}}}{(1-s)LP^t_{\mathrm{X}} + s(1-d) + (1-s)(1-L)(1-d)}.$$

When $P = 1$, similar to calculation in section A.2.1 the threshold for CMS fixation becomes

$$g = max\left\{1 + s - 2sd, (1-s)(2d-1) + \frac{4(1-d)}{\alpha(1-e_{\mathrm{m}})}\right\}. \tag{A9}$$

For general cases where the pollen limitation threshold is an arbitrary value P, the threshold for CMS fixation under pollen limitation is $g = (1-s)(2d-1) + \frac{4(1-d)P}{\alpha(1-e_{\mathrm{m}})}$.
