# Supplementary material for: The evolution and maintenance of trioecy with cytoplasmic male sterility
Source: Heredity (Edinb). 2024 Oct 14;134(1):1–9. doi: 10.1038/s41437-024-00729-7 (PMC11723941; doi:10.1038/s41437-024-00729-7)
Supplement: Supplementary file 2 — Output of the Mathematica script - Model 1 [file 41437_2024_729_MOESM2_ESM.pdf]

In[35]:=

$$x1n = x1 \frac{1}{x1\,(1 - s\,d) + x2\,g}\left(s\,(1-d) + (1-s)\frac{x1 + \frac{x3}{2}a + \frac{x4}{2}a\,(1-\epsilon)}{x1 + x3\,a + x4\,a\,(1-\epsilon)}\right);$$

(* Hermaphrodite *)

$$x2n = x2\,\frac{g}{x1\,(1 - s\,d) + x2\,g}\,\frac{x1 + \frac{x3}{2}a + \frac{x4}{2}a\,(1-\epsilon)}{x1 + x3\,a + x4\,a\,(1-\epsilon)};\ \text{(* Female *)}$$

$$x3n = x1\,\frac{1-s}{x1\,(1 - s\,d) + x2\,g}\,\frac{\frac{x3}{2}a + \frac{x4}{2}a\,(1-\epsilon)}{x1 + x3\,a + x4\,a\,(1-\epsilon)};\ \text{(* Male *)}$$

$$x4n = x2\,\frac{g}{x1\,(1 - s\,d) + x2\,g}\,\frac{\frac{x3}{2}a + \frac{x4}{2}a\,(1-\epsilon)}{x1 + x3\,a + x4\,a\,(1-\epsilon)};\ \text{(* Male with CMS *)}$$

In[39]:=

```
J = {{D[x1n, x1], D[x1n, x2], D[x1n, x3], D[x1n, x4]},
   {D[x2n, x1], D[x2n, x2], D[x2n, x3], D[x2n, x4]},
   {D[x3n, x1], D[x3n, x2], D[x3n, x3], D[x3n, x4]},
   {D[x4n, x1], D[x4n, x2], D[x4n, x3], D[x4n, x4]}}; (* Jacobian *)
```

In[40]:=

```
eig = Eigenvalues[J]; (* λ is the leading eigenvalue,
that is the largest numerically *)
λ = eig[[4]]; (* We need -1 < λ < 1 for the considered
 point to be stable (i.e., resistant to invasion) *)
```

In[6]:=

```
%4
```

In[7]:=

```
eig /. x1 → 0.25 /. x2 → 0.25 /. x3 → 0.25 /. x4 → 0.25 /. a → 4 /. s → 0.3 /. d → 0.1 /.
  g → 4 /. ϵ → 0.2

Assuming[a > 0 && 0 < d < 1 && 0 < s < 1 && 0 < ϵ < 1 && 0 < g ,
 eig[[4]] /. x1 → 1 /. x3 → 0 /. x2 → 0 /. x4 → 0 // Simplify] (* Condition
  for males and CMS not to invade hermpahroditism is this term < 1 *)
```

Out[12]=

$$\frac{a + 2\,g - a\,s + \text{Abs}[a - 2\,g - a\,s]}{4 - 4\,d\,s}$$

In[30]:=

$$\text{Reduce}\left[\frac{a + 2\,g - a\,s + \text{Abs}[a - 2\,g - a\,s]}{4 - 4\,d\,s} < 1\ \&\&\ 0 < d < 1\ \&\&\ 0 < s < 1\ \&\&\ a > 0\ \&\&\ g > 0\right]\ //\ \text{FullSimplify}$$

Out[30]=

$$s > 0\ \&\&\ d > 0\ \&\&\ g > 0\ \&\&\ a > 0\ \&\&\ s < 1\ \&\&\ d < 1\ \&\&\ g + d\,s < 1\ \&\&\ a + 2\,d\,s < 2 + a\,s$$

```
(* Condition for CMS not to invade hermpahroditism g+d s<1 ,
Condition for males not to invade hermpahroditism a+2 d s<2+a s *)

Solve[{x1n == x1 /. x2 → 0 /. x4 → 0, x3n == x3 /. x2 → 0 /. x4 → 0}, {x1, x3}] //
 Simplify(* Androdioecious equilibria
  frequencies when CMS cannot invade but males did *)
```

Out[26]=

$$\left\{\{x1 \to 1,\ x3 \to 0\},\ \left\{x1 \to \frac{a\,(-1 + (-1 + 2\,d)\,s)}{2\,(-1 + a)\,(-1 + d\,s)},\ x3 \to \frac{2 + a\,(-1 + s) - 2\,d\,s}{2\,(-1 + a)\,(-1 + d\,s)}\right\}\right\}$$

In[31]:=
```
Assuming[a + 2 d s < 2 + a s && 0 < d < 1 && 0 < s < 1 && 0 < ϵ < 1 && 0 < g,
  λ /. x1 → (a (-1 + (-1 + 2 d) s))/(2 (-1 + a) (-1 + d s)) /. x2 → 0 /. x3 → (2 + a (-1 + s) - 2 d s)/(2 (-1 + a) (-1 + d s)) /. x4 → 0 //
   Simplify]
  (* Condition for CMS not invaded androdioecy are these terms < 1 *)
```

Out[31]=

$$\begin{cases} \frac{2-2 d s}{a-a s} & !\left(a < 0 \veebar a g (-1+s) + 2 s \left(1 - g + d (-3+g-s) + 2 d^2 s\right) < -2\right) \\ \frac{g (a - a s - 2 (-1+d) s)}{a (-1+s) (-1+(-1+2 d) s)} & \text{True} \end{cases}$$

In[34]:=
```
Reduce[
  (g (a - a s - 2 (-1 + d) s))/(a (-1 + s) (-1 + (-1 + 2 d) s)) < 1 && 0 < d < 1 && 0 < s < 1 && a + 2 d s > 2 + a s && 0 < g]
 (* Condition for CMS NOT to invade androdioecious population *)
```

Out[34]=

$$0 < d < 1 \,\&\&\, a > 2 \,\&\&\, 0 < s < \frac{-2+a}{a-2 d} \,\&\&\, 0 < g < \frac{-a + 2 a d s + a s^2 - 2 a d s^2}{-a - 2 s + a s + 2 d s}$$

In[25]:=
```
Assuming[a > (2 (-1 + d s))/(-1 + s) && 0 < d < 1 && 0 < s < 1 && 0 < ϵ < 1 && 0 < g,
  eig〚4〛 /. x1 → 0 /. x3 → 0 /. x2 → 1 / 2 /. x4 → 1 / 2 // Simplify]
 (* Condition for CMS fixation is this term < 1 *)
```

Out[25]=

$$\frac{1 + s - 2 d s}{g}$$

```
(* Condition for CMS fixation (1+s-2 d s)/g < 1 *)
```
