# Supplementary material for: The evolution and maintenance of trioecy with cytoplasmic male sterility
Source: Heredity (Edinb). 2024 Oct 14;134(1):1–9. doi: 10.1038/s41437-024-00729-7 (PMC11723941; doi:10.1038/s41437-024-00729-7)
Supplement: Supplementary file 3 — Output of the Mathematica script - Model 2 [file 41437_2024_729_MOESM3_ESM.pdf]

In[•]:=

```
L = Min[a x3 + a (1 - ϵ) x4 + x1, 1] ;
```

$$x1n = x1 \frac{1}{x1\,(L - L\,s + s - s\,d) + L\,x2\,g} \left( s\,(1 - d) + L\,(1 - s) \frac{x1 + \frac{x3}{2} a + \frac{x4}{2} a\,(1 - \epsilon)}{x1 + x3\,a + x4\,a\,(1 - \epsilon)} \right);$$

```
(* Hermaphrodite *)
```

$$x2n = x2 \frac{L\,g}{x1\,(L - L\,s + s - s\,d) + L\,x2\,g} \frac{x1 + \frac{x3}{2} a + \frac{x4}{2} a\,(1 - \epsilon)}{x1 + x3\,a + x4\,a\,(1 - \epsilon)};\ \text{(* Female *)}$$

$$x3n = x1 \frac{L\,(1 - s)}{x1\,(L - L\,s + s - s\,d) + L\,x2\,g} \frac{\frac{x3}{2} a + \frac{x4}{2} a\,(1 - \epsilon)}{x1 + x3\,a + x4\,a\,(1 - \epsilon)};\text{(* Male *)}$$

$$x4n = x2 \frac{L\,g}{x1\,(L - L\,s + s - s\,d) + L\,x2\,g} \frac{\frac{x3}{2} a + \frac{x4}{2} a\,(1 - \epsilon)}{x1 + x3\,a + x4\,a\,(1 - \epsilon)};\text{(* Male with CMS *)}$$

In[•]:=

```
J = {{D[x1n, x1], D[x1n, x2], D[x1n, x3], D[x1n, x4]},
    {D[x2n, x1], D[x2n, x2], D[x2n, x3], D[x2n, x4]},
    {D[x3n, x1], D[x3n, x2], D[x3n, x3], D[x3n, x4]},
    {D[x4n, x1], D[x4n, x2], D[x4n, x3], D[x4n, x4]}}; (* Jacobian *)
```

In[•]:=

```
eig = Eigenvalues[J]; (* λ is the leading eigenvalue,
that is the largest numerically *)
λ = eig⟦4⟧; (* We need -1 < λ < 1 for the considered
 point to be stable (i.e., resistant to invasion) *)
```

In[•]:=

```
eig /. x1 → 0.25 /. x2 → 0.25 /. x3 → 0.25 /. x4 → 0.25 /. a → 5 /. s → 0.3 /. d → 0.1 /.
  g → 1.3 /. ϵ → 0.2
```

Out[•]=

```
{0, 0., 0.163395, 1.16813}
```

```
Assuming[a > 0 && 0 < d < 1 && 0 < s < 1 && 0 < ϵ < 1 && 0 < g ,
 eig⟦4⟧ /. x1 → 1 /. x3 → 0 /. x2 → 0 /. x4 → 0 // Simplify] (* Condition
  for males and CMS not to invade hermpahroditism is this term < 1 *)
```

Out[•]=

$$\frac{1}{2\,(-1 + d\,s)^2} \text{Root}\left[\left(2\,a\,g - 2\,a\,g\,s - 4\,a\,d\,g\,s + 4\,a\,d\,g\,s^2 + 2\,a\,d^2\,g\,s^2 - 2\,a\,d^2\,g\,s^3\right) \#1 + \left(-a - 2\,g + a\,s + a\,d\,s + 2\,d\,g\,s - a\,d\,s^2\right) \#1^2 + \#1^3\,\&,\ 3\right]$$

In[•]:=

$$\text{Reduce}\left[\frac{1}{2\,(-1 + d\,s)^2} \text{Root}\left[\left(2\,a\,g - 2\,a\,g\,s - 4\,a\,d\,g\,s + 4\,a\,d\,g\,s^2 + 2\,a\,d^2\,g\,s^2 - 2\,a\,d^2\,g\,s^3\right) \#1 + \left(-a - 2\,g + a\,s + a\,d\,s + 2\,d\,g\,s - a\,d\,s^2\right) \#1^2 + \#1^3\,\&,\ 3\right] < 1\ \&\&\ 0 < d < 1\ \&\&\ 0 < s < 1\ \&\&\ a > 0\ \&\&\ g > 0\right]\ //\ \text{FullSimplify}$$

Out[•]=

```
s > 0 && d > 0 && g > 0 && a > 0 && s < 1 && d < 1 && g + d s < 1 && a + 2 d s < 2 + a s
```

```
(* Condition for CMS not to invade hermpahroditism: g + d s < 1 ,
Condition for males not to invade hermpahroditism: a + 2 d s < 2 + a s *)
```

```
Assuming[a > 2 (-1 + d s)/(-1 + s) && 0 < d < 1 && 0 < s < 1 && 0 < ϵ < 1 && 0 < g,
 eig[[4]] /. x1 → 0 /. x3 → 0 /. x2 → 1 / 2 /. x4 → 1 / 2 // Simplify]
(* Condition for CMS fixation is this term < 1 *)
```

Out[ ]=

$$\left(8\,\text{Root}\left[\left(-2 a^2 g s \,\text{Min}\left[1, \frac{1}{2} a (1-\epsilon)\right] + 2 a^2 d g s \,\text{Min}\left[1, \frac{1}{2} a (1-\epsilon)\right] + 4 a^2 g s \epsilon \,\text{Min}\left[1, \frac{1}{2} a (1-\epsilon)\right] - 4 a^2 d g s \epsilon \,\text{Min}\left[1, \frac{1}{2} a (1-\epsilon)\right] - 2 a^2 g s \epsilon^2 \,\text{Min}\left[1, \frac{1}{2} a (1-\epsilon)\right] + 2 a^2 d g s \epsilon^2 \,\text{Min}\left[1, \frac{1}{2} a (1-\epsilon)\right] - a^2 g \,\text{Min}\left[1, \frac{1}{2} a (1-\epsilon)\right]^2 + a^2 g s \,\text{Min}\left[1, \frac{1}{2} a (1-\epsilon)\right]^2 + 2 a^2 g \epsilon \,\text{Min}\left[1, \frac{1}{2} a (1-\epsilon)\right]^2 - 2 a^2 g s \epsilon \,\text{Min}\left[1, \frac{1}{2} a (1-\epsilon)\right]^2 - a^2 g \epsilon^2 \,\text{Min}\left[1, \frac{1}{2} a (1-\epsilon)\right]^2 + a^2 g s \epsilon^2 \,\text{Min}\left[1, \frac{1}{2} a (1-\epsilon)\right]^2\right) \#1^2 + 8 \#1^3 \&, 3\right]\right) \Big/ \left(a^2 g^2 (-1+\epsilon)^2 \,\text{Min}\left[1, -\frac{1}{2} a (-1+\epsilon)\right]^2\right)$$

```
Reduce[
 (8 Root[(-2 a^2 g s Min[1, 1/2 a (1 - ϵ)] + 2 a^2 d g s Min[1, 1/2 a (1 - ϵ)] + 4 a^2 g s ϵ Min[
          1, 1/2 a (1 - ϵ)] - 4 a^2 d g s ϵ Min[1, 1/2 a (1 - ϵ)] -
         2 a^2 g s ϵ^2 Min[1, 1/2 a (1 - ϵ)] + 2 a^2 d g s ϵ^2 Min[1, 1/2 a (1 - ϵ)] -
         a^2 g Min[1, 1/2 a (1 - ϵ)]^2 + a^2 g s Min[1, 1/2 a (1 - ϵ)]^2 +
         2 a^2 g ϵ Min[1, 1/2 a (1 - ϵ)]^2 - 2 a^2 g s ϵ Min[1, 1/2 a (1 - ϵ)]^2 - a^2 g ϵ^2
          Min[1, 1/2 a (1 - ϵ)]^2 + a^2 g s ϵ^2 Min[1, 1/2 a (1 - ϵ)]^2) #1^2 + 8 #1^3 &, 3]) /
    (a^2 g^2 (-1 + ϵ)^2 Min[1, -1/2 a (-1 + ϵ)]^2) < 1 && 0 < d < 1 && 0 <
    s < 1 && a > 2 (-1 + d s)/(-1 + s) && 0 <
    ϵ <
    1 && 0 <
    g,
  g] // Simplify (* Condition
  for
  CMS
  fixation *)
```

Out[•]= $0 < d < 1 \,\&\&\, 0 < s < \frac{-2+a}{a-2d} \,\&\&\, 0 < \epsilon < 1 \,\&\&$
$\left(\left(a\,(-4\,(-1+d)\,s + a\,(-1+g+s)\,(-1+\epsilon))\,(-1+\epsilon) > 0 \,\&\&\, 2 < a \leq -\frac{2}{-1+\epsilon}\right) \,||\, \left(a > -\frac{2}{-1+\epsilon} \,\&\&\, g > 1 + s - 2\,d\,s\right)\right)$

```
(* Condition for CMS fixation if the dioecious
  population is not pollen limited g > 1 + s - 2 d s,
if the dioecious population is pollen limited a
  (-4 (-1+d) s+a (-1+g+s) (-1+ϵ)) (-1+ϵ)>0 or,
equivalently, a (-1+g+s) (-1+ϵ)<4 (-1+d) s *)

(* We do not analyse the condition for CMS not to invade androdioecy for 2
  reasons. (1) It is the same as in the case of no pollen limitation -
 model 1, because no pollen limitation should arise in our model if
 females did not invade and all plants could produce pollen. Hence,
this condition is already analysed in the script of model
 1. (2) The analysis of the condition for CMS to invade
 androdioecy takes too much time to run because estimating
 equilibrium frequencies is too complicated for this model *)
```
